# Supplementary material for: Chromothripsis during telomere crisis is independent of NHEJ, and consistent with a replicative origin
Source: Genome Res. 2019 May;29(5):737–49. doi: 10.1101/gr.240705.118 (PMC6499312; doi:10.1101/gr.240705.118)
Supplement: Supplemental Material [file supp_gr.240705.118_Supplemental_file_1.zip › contigs/annotated_contigs/DB67/contig.2.DB67_length_583_mean_cov_6.71183533448.docx]

**DB67_length_583_mean_cov_6.71183533448**

```
GTGGTGGCTGGGGCCTGTAGTCCCAGCTACTCAGGAGGCTGAGGAAAGAGAATGGCCTGAACCCGGGAGGCGGAGTTTGCAGTGAGCCG
 >chr7:72788621-72789033 + E=1e-149 p=1e-02
AGATCGTGCCACTGCACTCCAGCCTGAGTGACAGAGCGAGATCCCGTCTCAAAAAAAAAAAAATTTAAACCAGATCTAAAGAGAGGCCT

GGCGTGGTGGCTCACGCCTGTAATTCCAGCACTTTGGGAGGCCAAGGCGGGCAGATCACCTGAGGTCAGGAGTTCGAGACCAGCCTGGC

CAACATGGCCAAACCCCGTCTCTACTAAAAATACAAAAATCAGCCGGGCGTCATGTTGGGCGCCTGTAATCCCAGTTACTAGAGAGGCT

GAGGCAGGAGAATCACTTGAACCTGGGAGGCGGAGGTTGCAGTGAGCTGAGAT|GGAGCCA|TTGTAAATAAAAGGCTGAGATGGAAAC
                                                             >chr5:171622524-171622691
GCAGGGCTCTCTGCCTGCAAAGCCCATGCTGAGTCTCTCTGGGATGCTTGAGGAGTTTAAGCTCCCTGGTAACCTCCCTGCTGATGCAG
+ E=8e-89
TACCCAGGAGGTAACGCCATGCTCATGTCTTCTGGTCACAGAGGGACTGGA
```
